# Supplementary material for: Proton and molecular permeation through the basal plane of monolayer graphene oxide
Source: Nat Commun. 2023 Nov 27;14:7756. doi: 10.1038/s41467-023-43637-w (PMC10682477; doi:10.1038/s41467-023-43637-w)
Supplement: Supplementary file 1 — Supplementary Information [file 41467_2023_43637_MOESM1_ESM.pdf]

Supplementary Information

Proton and molecular permeation through the basal plane of monolayer graphene oxide

Wu et al

This file contains:

• Supplementary Figures 1-7

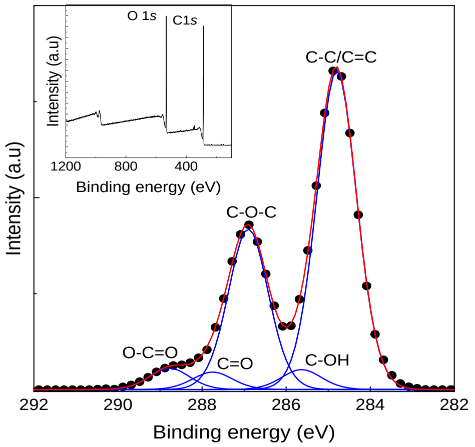

Supplementary Figure 1| High resolution XPS spectra of C1s region of GO. The filled circles and red curve are the raw data and their fitting with a multipeak function, respectively. The fitting required five components (blue curves) representing the energy shifts of photoelectrons due to different bonding environments. The data show that C-O-C was the dominant functional group on the GO films. Inset: XPS survey spectrum of the same sample showing C1s and O1s regions.

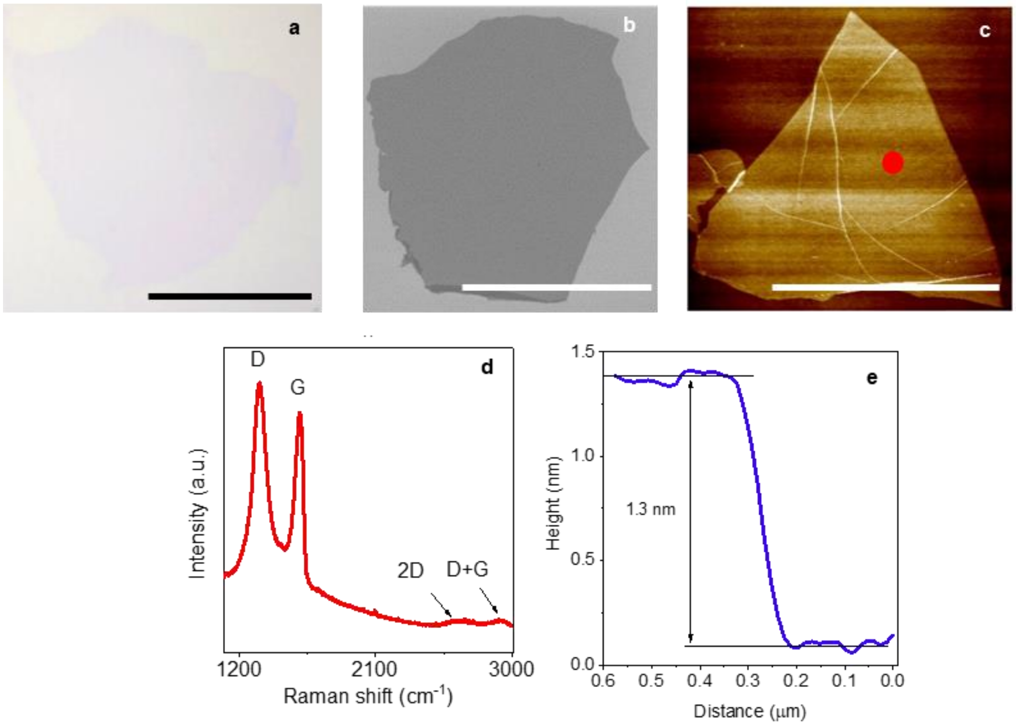

Supplementary Figure 2| Characterization of monolayer GO. **a**, **b** and **c**, Optical, electron and AFM micrographs, respectively, of representative GO monolayers used in our studies. Scale bars, 20 μm. The red circle in panel c marks the area of 2 μm in diameter, which was probed in our experiments using helium-leak detectors. **d**, Typical Raman spectrum measured for our GO monolayers. **e**, Height profile for a typical GO monolayer flake on a silicon oxide wafer.

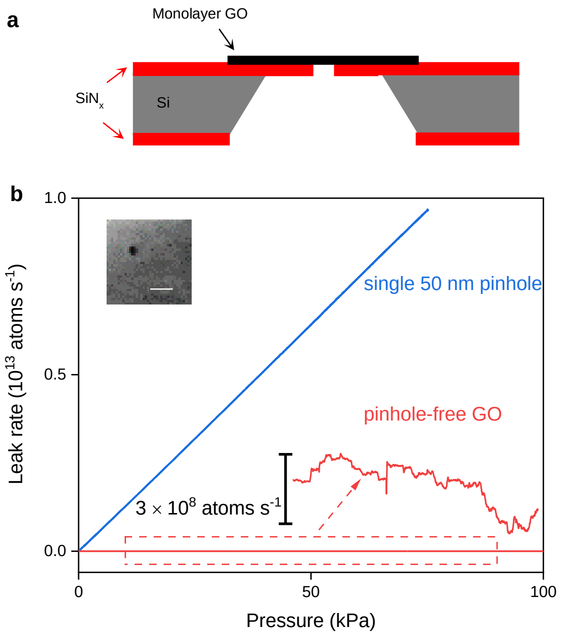

Supplementary Figure 3| Impermeability of monolayer GO to helium atoms. **a**, Schematic of monolayer GO devices used for measurements by the helium-leak detector. **b**, Leak rates through a monolayer membrane with a single pinhole of ~50 nm in diameter as a function of applied He pressure (blue curve). Inset, SEM image of the pinhole. Scale bar, 100 nm. Red curve: Corresponding leak rate for a GO membrane of 2 μm in diameter without defects visible in AFM. The inset magnifies the red curve showing its background noise level that determines our detection limit as a few 10^8 atoms per second.

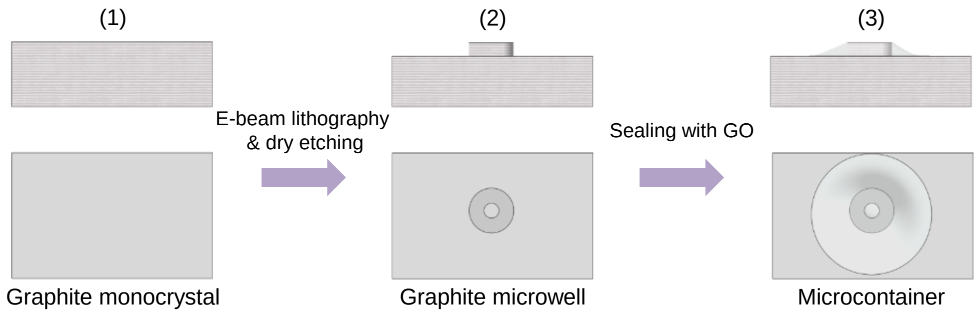

Supplementary Figure 4| Fabrication of microcontainer devices. Top row, side view. Bottom row, top view. The graphite monocrystal (1) was etched to yield a micrometer-size well (2) and then sealed with monolayer GO (3).

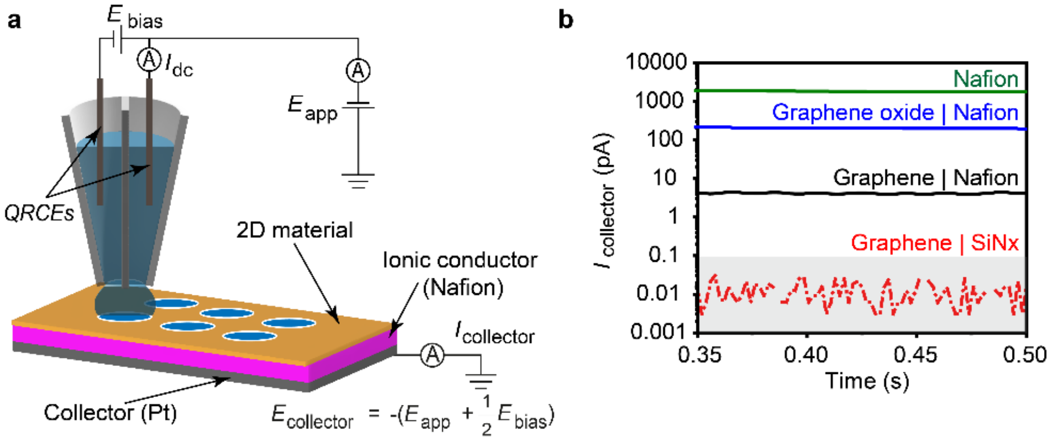

Supplementary Figure 5| Scanning electrochemical cell microscopy. a, Schematic of our SECCM setup. A pipette with a nanometer-size tip contains two quasi-reference counter electrodes (QRCE) inside two reservoirs filled with an HCl electrolyte. The voltage E_{bias} between the QRCEs yields I_{dc} that is used as a feedback signal. The voltage E_{app} applied to one of the QRCEs determines the overall potential used to inject protons. During the scanning protocol the probe approaches the crystal, makes contact over the area marked by the blue circle and is then retracted and moved to another position (different contact areas marked with blue circles). **b**, Steady state SECCM currents collected from a device without any crystal covering Nafion (green); monolayer GO on top of Nafion (blue); monolayer graphene on Nafion (black); and the same graphene monolayer on top of silicon-nitride away from the aperture (red).

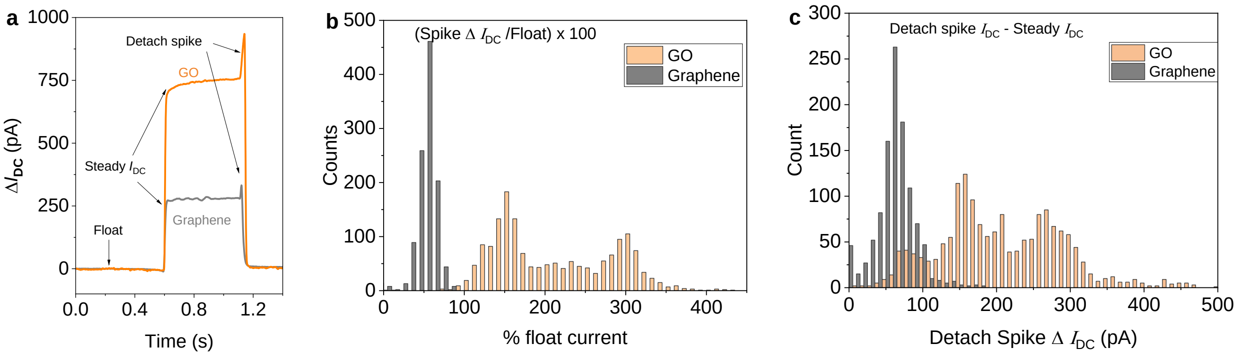

Supplementary Figure 6| Meniscus wetting in GO and graphene membranes. a, Examples of ΔI_{DC} - t characteristics (current between the two electrodes in the nanopipette) for graphene (grey) and GO (orange). The profiles display three stages. Initially, the probe is not in contact with the sample ('float' stage). The 'float' current is subtracted from I_{DC} to yield $\Delta I_{DC} = I_{DC} - I_{DC}^{float}$. In this 'float' stage, then, $\Delta I_{DC} = 0$. In the second stage, the probe is in contact with the sample yielding $\Delta I_{DC}^{Steady} > 0$. In the third stage, the probe retracts and ΔI_{DC} displays a 'detach spike' before dropping back to the float level. **b**, The size of the meniscus can be qualitatively estimated from the ΔI_{DC} current because this current depends on meniscus size. For each approach-retract cycle (each at a different area in the sample), we extract %float current = $\Delta I_{DC}^{Steady} / I_{DC}^{float}$ x100. The figure shows statistics of this quantity for graphene (grey) and GO (orange). The latter distribution shows larger values and a broader distribution, consistent with a larger meniscus and a heterogeneous distribution of the oxidised sites. **c**, The 'detach spike' in the ΔI_{DC} provides an independent estimate of the meniscus size. For each approach-retract cycle we calculate Detach Spike $\Delta I_{DC} = \Delta I_{DC}^{Detach\text{-}spike}$ - ΔI_{DC}^{Steady}. The statistics for this quantity mirror those in panel **b**.

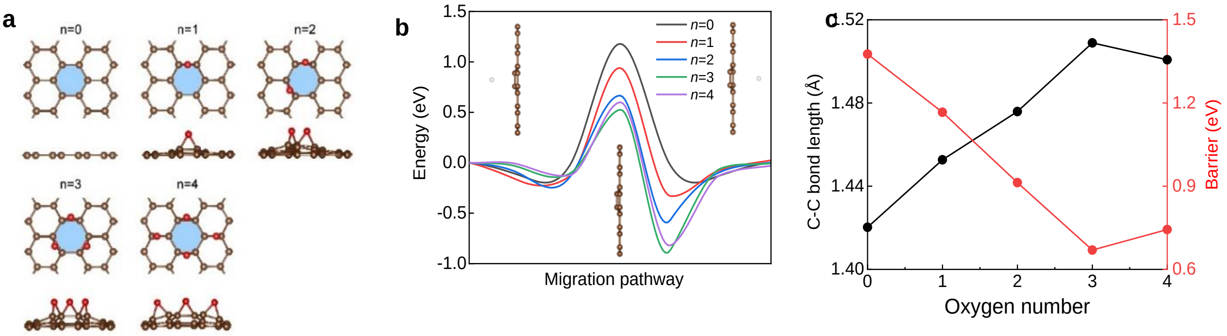

Supplementary Figure 7| Density functional theory calculations. a, Atomic structures for graphene with different numbers of epoxy groups. The blue areas mark the hexagonal ring onto which the epoxy groups are bonded. The brown and red balls represent C and O atoms, respectively. **b**, Energy profiles for proton transport through the oxidized hexagonal ring. Each curve represents the profile of a graphene-epoxy structure with different number of epoxy groups (colour coded). Insets: schematics of atomic configurations for the initial, maximum energy and final states for the proton transport process (from left to right). The white balls denote protons. **c**, Calculated average C-C bond length of the graphene lattice (left axis, black) and permeation barrier for protons (right, red) as a function of the number of bonded oxygen atoms.
